# Data on urinary pteridines as a discriminator of atherosclerotic risk in patients with diabetes

**DOI:** 10.1016/j.dib.2022.107897

**Published:** 2022-02-02

**Authors:** Mikio Marumo, Kazumi Ekawa, Ichiro Wakabayashi

**Affiliations:** Department of Environmental and Preventive Medicine, Hyogo College of Medicine, Nishinomiya, Japan

**Keywords:** Alcohol, Diabetes, Oxidative stress, Pteridines, Smoking

## Abstract

Presented here are the supplemental data of the research article “Urinary pteridines as a discriminator of atherosclerotic risk in patients with diabetes” [1]. These data provide the first information on variables that affect urinary levels of pteridines (oxidized-form pteridine derivatives) in patients with diabetes mellitus. In linear regression analysis, gender (women vs. men), current history of smoking and urinary albumin showed significant positive correlations with pteridines, while there were significant inverse correlations of pteridines with a history of alcohol drinking and body mass index. The above associations were confirmed by using analysis of covariance and logistic regression analysis. Among the eight variables (age, gender, medication therapy for diabetes, smoking, alcohol drinking, body mass index, hemoglobin A_1c_ and urinary albumin) tested, smoking showed the strongest association with urinary pteridines.

## Specifications Table

**Table:** 

Subject	Health and medical sciences
Specific subject area	Cardiology and Cardiovascular Medicine
Type of data	Table
How data were acquired	Data were cross-sectionally collected and analyzed. Calculations were conducted with IBM SPSS Statistics for Windows, Version 25.0. (Armonk, NY, USA).
Data format	Raw Analyzed
Description of data collection	Data were collected from outpatients with type 2 diabetes in Kobe Tokushukai Hospital in Hyogo Prefecture in Japan.
Data source location	Institution: Hyogo College of Medicine City/Town/Region: Nishinomiya, Hyogo Country: Japan
Data accessibility	With the article
Related research article	M. Marumo, K. Ekawa, I. Wakabayashi, Urinary pteridines as a discriminator of atherosclerotic risk in patients with diabetes, Atherosclerosis Plus 46 (2021): 27-34. doi.org/10.1016/j.athplu.2021.11.001.

## Value of the Data

• These data provide the information on associations of urinary pteridines with gender, smoking, alcohol drinking, body mass index, and urinary albumin in patients with diabetes.
• These data support our recent finding that urinary pteridine level is a new biomarker of oxidative stress.
• These data are useful when considering correction of the lifestyle of patients with diabetes for reducing oxidative stress that is an important pathogenesis of complicated cardiovascular diseases.
• These data show the usefulness of urinary pteridine level to evaluate oxidative stress in smokers with diabetes.

## 1 Data Description

We chose eight variables, including age, gender, current history of medication therapy for diabetes, current history of smoking, current history of alcohol drinking, body mass index (BMI), hemoglobin A_1c_ level and urinary albumin level, to investigate their relationships with urinary pteridine level, which has been proposed to be an oxidative stress marker in a general population [2] and has been shown to be associated with blood coagulation and atherosclerosis in the lower extremities of patients with diabetes [1].

Table 1 shows the results of linear regression analysis. Urinary pteridine levels were used after logarithmic transformation in order to make them show a normal distribution. In multivariable analysis, the correlation of each variable with creatinine-corrected pteridines after logarithmic transformation was analyzed with adjustment for the other seven variables. Both in univariable analysis and multivariable analysis, gender (women vs. men), smoking and urinary albumin showed significant positive correlations with pteridines, while alcohol drinking and BMI were inversely correlated with pteridines.

**Table 1:** Correlations of age, gender, therapy for diabetes, smoking, alcohol drinking, BMI, hemoglobin A_1c_ and urinary albumin with urinary pteridines.

	Univariable	Multivariable
Age	‒0.011	‒0.013
Gender (women/men)	0.132*	0.176*
Therapy for diabetes	0.052	0.015
Smoking	0.141*	0.260**
Alcohol drinking	‒0.181**	‒0.155*
Body mass index	‒0.158*	‒0.159*
Hemoglobin A_1c_	‒0.042	0.046
Urinary albumin	0.183**	0.141*

Shown are Spearman's rank correlation coefficients in univariable analysis and standardized partial regression coefficients in multivariable analysis. Pteridine levels were corrected by urinary creatinine levels and used for analysis after logarithmic transformation. In multivariable analysis, the other seven variables from the eight variables tested were adjusted. Symbols indicate significant correlations (*, *p* < 0.05; **, *p* < 0.01).

Next, mean levels of log-transformed pteridines were compared in different groups of each variable (Table 2). In univariable analysis, pteridine levels were significantly lower in regular drinkers than in nondrinkers. Also in univariable analysis, levels of pteridines were higher with a marginal significance in women than in men, in heavy smokers than in nonsmokers and in the 3rd tertile group of urinary albumin than in its 1st tertile group. In multivariable analysis, pteridine levels were significantly higher in women than in men and in heavy smokers than in nonsmokers, and were significantly lower in the 3rd tertile group of BMI than in its 1st tertile group. Also in multivariable analysis, there was marginal significance in the lower mean level of pteridines in regular drinkers than in nondrinkers and in the higher mean level of pteridines in the 3rd tertile group of urinary albumin than in the 1st tertile group.

**Table 2:** Comparisons of urinary pteridine levels in different groups of age, gender, therapy for diabetes, smoking, alcohol drinking, BMI, hemoglobin A_1c_ and urinary albumin.

	Univariable	Multivariable
Age		
1st tertile	1.173 (1.12*9*-1.217)	1.166 (1.124-1.207)
2nd tertile	1.148 (1.108-1.188)	1.164 (1.123-1.204)
3rd tertile	1.171 (1.127-1.214)	1.162 (1.119-1.205)
Gender		
Men	1.147 (1.114-1.179)	1.136 (1.105-1.168)
Women	1.191 (1.156-1.227)^#^	1.208 (1.166-1.249)*
Therapy for diabetes		
None	1.214 (1.128-1.301)	1.210 (1.133-1.287)
Oral antidiabetic drugs	1.146 (1.119-1.173)	1.150 (1.124-1.177)
Insulin	1.228 (1.159-1.297)	1.207 (1.142-1.271)
Smoking		
Nonsmokers	1.146 (1.118-1.173)	1.132 (1.103-1.160)
Light smokers	1.187 (1.103-1.270)	1.209 (1.127-1.290)
Heavy smokers	1.218 (1.157-1.279)^##^	1.257 (1.201-1.313)**
Alcohol drinking		
Nondrinkers	1.198 (1.164-1.232)	1.195 (1.163-1.227)
Occasional drinkers	1.115 (1.044-1.186)	1.112 (1.044-1.180)
Regular drinkers	1.124 (1.086-1.162)*	1.131 (1.089-1.172)^###^
Body mass index		
1st tertile	1.193 (1.150-1.236)	1.193 (1.152-1.233)
2nd tertile	1.168 (1.127-1.209)	1.179 (1.139-1.219)
3rd tertile	1.130 (1.087-1.172)	1.119 (1.078-1.159)*
Hemoglobin A_1c_		
1st tertile	1.188 (1.141-1.234)	1.170 (1.129-1.211)
2nd tertile	1.154 (1.116-1.192)	1.164 (1.124-1.203)
3rd tertile	1.149 (1.107-1.192)	1.157 (1.114-1.200)
Urinary albumin		
1st tertile	1.134 (1.088-1.180)	1.139 (1.099-1.179)
2nd tertile	1.152 (1.110-1.194)	1.149 (1.109-1.190)
3rd tertile	1.205 (1.168-1.243)^####^	1.203 (1.163-1.243)^#####^

Shown are means of pteridine levels with 95% confidence intervals in parentheses. Pteridine levels were corrected by urinary creatinine levels and used for analysis after logarithmic transformation. In multivariable analysis (ANCOVA), the other seven variables from the eight variables tested were adjusted. Symbols indicate significant differences (*, *p* < 0.05; **, *p* < 0.01) and marginally significant differences (^#^, *p* = 0.077; ^##^, *p* = 0.065; ^###^*p* = 0.062; ^####^, *p* = 0.061; ^#####^, *p* = 0.089) from each reference (men, no therapy, nonsmokers, nondrinkers or 1st tertile).

The results of logistic regression analysis for the relationship of each variable with high levels of urinary pteridines are shown in Table 3. The cutoff value of high levels of urinary pteridines used in this study was 15.8 μM/g creatinine, which was determined by ROC analysis using both low ankle-brachial index and high d-dimer as the endpoint in patients with diabetes [1]. In multivariable analysis, the odds ratio for high levels of pteridines was significantly high in women when men were the reference [odds ratio: 2.18 (1.14-4.19)]. When nonsmokers were the reference, the odds ratio for high levels of pteridines was significantly high in heavy smokers [odds ratio: univariable analysis, 2.14 (1.15-4.00); multivariable analysis, 4.06 (1.85-8.89)]. Both in univariable analysis and multivariable analysis, occasional and regular drinkers showed low odds ratios for high levels of pteridines when nondrinkers were the reference. In multivariable analysis, the odds ratio for high levels of pteridines was significantly low in the 3rd tertile group of BMI when the 1st tertile was the reference. The odds ratio for high levels of pteridines was high in the 3rd tertile group of urinary albumin when the 1st tertile was the reference (odds ratio: univariable analysis, 1.90 (1.02-3.54); multivariable analysis, 1.92 (0.99-3.71)].

**Table 3:** Odds ratios for high levels of urinary pteridines (≥ 15.8 μM/g creatinine) of each group vs. its reference of age, gender, therapy for diabetes, smoking, alcohol drinking, BMI, hemoglobin A_1c_ and urinary albumin.

	Univariable	Multivariable
Age		
1st tertile	1.00	1.00
2nd tertile	0.94 (0.51-1.74)	1.31 (0.65-2.65)
3rd tertile	0.90 (0.48-1.67)	0.95 (0.46-1.99)
Gender		
Men	1.00	1.00
Women	1.52 (0.91-2.55)	2.18 (1.14-4.19)*
Therapy for diabetes		
None	1.00	1.00
Oral drugs	0.62 (0.27-1.47)	0.63 (0.25-1.58)
Insulin	1.06 (0.38-2.98)	1.46 (0.35-6.15)
Smoking		
None	1.00	1.00
Light	1.06 (0.43-2.63)	1.34 (0.46-3.90)
Heavy	2.14 (1.15-4.00)*	4.06 (1.85-8.89)**
Alcohol drinking		
None	1.00	1.000
Occasional	0.31 (0.12-0.79)*	0.24 (0.09-0.68)**
Regular	0.54 (0.30-0.95)*	0.56 (0.29-1.06)^#^
Body mass index		
1st tertile	1.00	1.00
2nd tertile	0.91 (0.49-1.67)	1.00 (0.52-1.92)
3rd tertile	0.61 (0.33-1.15)	0.48 (0.24-0.98)*
Hemoglobin A_1c_		
1st tertile	1.00	1.00
2nd tertile	0.88 (0.48-1.60)	1.21 (0.62-2.36)
3rd tertile	0.67 (0.36-1.26)	0.82 (0.40-1.70)
Urinary albumin		
1st tertile	1.00	1.00
2nd tertile	1.13 (0.60-2.14)	1.06 (0.54-2.10)
3rd tertile	1.90 (1.02-3.54)*	1.92 (0.99-3.71)^##^

Shown are odds ratios with their 95% confidence intervals. In multivariable analysis, the other seven variables from the eight variables tested were adjusted. Symbols indicate significant and marginally significant odds ratios (*, *p* < 0.05; **, *p* < 0.01; #, *p* = 0.074; ##, *p* = 0.054).

## 2 Experimental Design, Materials and Methods

### 2.1 Study population

The subjects of this study were 257 outpatients (158 men and 99 women) with type 2 diabetes mellitus. The protocol of this study was approved by the ethics committees of Kobe Tokushukai Hospital (number: TGE00313-014) and Hyogo College of Medicine (number: 1766). Histories of medication therapy and lifestyles including cigarette smoking and alcohol drinking of each subject were surveyed by questionnaires. The subjects were divided by the frequency of current average alcohol drinking into three groups (nondrinkers, never; occasional drinkers, less than 2 days per week; regular drinkers, 2 days or more per week). The subjects were also divided into three groups by amount of current average cigarette consumption (nonsmokers, none; light smokers, < 20 cigarettes per day; heavy smokers, ≥ 20).

### 2.2 Measurements

At a health checkup, height and body weight of each subject were measured wearing light clothes, and BMI was calculated dividing weight in kilograms by the square of height in meters.

Fasting blood of each subject was collected in the morning, and plasma samples were obtained after centrifugation and were stored at ‒20 °C. Plasma hemoglobin A_1c_ level was measured using an automatic glycol-hemoglobin analyzer based on high-performance liquid chromatography. Using the formula prepared by the JDS (Japan Diabetes Society), hemoglobin A_1c_ values in plasma were standardized according to values of NGSP (National Glycohemoglobin Standardization Program) as follows [3]: hemoglobin A_1c_ (NGSP) (%) = 1.02 x hemoglobin A_1c_ (JDS) (%) + 0.25(%). Subjects were diagnosed as diabetes when they were receiving drug therapy for diabetes and/or they showed high plasma hemoglobin A_1c_ levels of 6.5% or higher, according to the criteria by the American Diabetes Association [4]. Urinary albumin concentrations were measured by immune-nephelometry using an auto-analyzer (JCA-BM8000 series, JEOL Ltd., Tokyo, Japan). Creatinine concentrations in urine were measured by the Jaffe method using a commercial kit (LabAssay^TM^ Creatinine [catalog number: 636-51011], FUJIFILM Wako Pure Chemical Corporation, Osaka, Japan).

Pteridines are aromatic chemical compounds and contain a wide variety of substitutions on the heterocyclic structure. In this study, levels of oxidized-form pteridine derivatives, including neopterin, biopterin, xanthopterin, isoxanthopterin, pterin and pterin-6-carboxylic acid, in urine were estimated by spectrofluorometry [2]. The excitation and emission wavelengths used in the spectrofluorometry were 360 nm and 450 nm, respectively.

### 2.3 Statistical analysis

A computer software program (IBM SPSS Statistics for Windows, Version 25.0. Armonk, NY: IBM Corp) was used for statistical analyses. In order to investigate the relationships of urinary pteridine levels with various factors including age, gender, therapy for diabetes, histories of smoking and alcohol drinking, BMI, hemoglobin A_1c_ and urinary albumin, we used three different analyses: correlations (correlation analysis), comparison of mean (analysis of variance [ANOVA] and analysis of covariance [ANCOVA]), and comparison of frequency (logistic regression analysis) as below.

For evaluation of correlations, Spearman's rank correlation coefficients and standardized partial regression coefficients were calculated in univariable analysis and multivariable linear regression analysis, respectively. In comparisons of means and frequencies, the values of each of age, BMI, hemoglobin A_1c_ level and urinary albumin level were arranged in ascending order; then the subjects were divided into three tertile groups of approximately equal sizes. The means of each variable were compared in the groups of each variable using Student's t test (two groups) or analysis of variance (ANOVA) followed by Scheffé’s F-test (three groups) in univariable analysis. Analysis of covariance (ANCOVA) followed by Student's t-test after Bonferroni correction was used to compare mean levels in multivariable analysis. In logistic regression analysis, the odds ratios for high levels of urinary pteridines in each group of the variables vs. its reference group were estimated before and after adjustment for the other seven explanatory variables except for each main explanatory variable from the eight variables tested. Since urinary pteridine and albumin levels did not show a normal distribution, their logarithmic transformation with a base of 10 was necessary to normalize them for performing parametric analyses including linear regression analysis, ANOVA and ANCOVA. Probability (*p*) values less than 0.05 were defined as significant.

## Ethics Statement

This study was conducted according to the principles of the Declaration of Helsinki. The protocol of this study was approved by the Ethics Committee of Kobe Tokushukai Hospital (number: TGE00313-014) and Hyogo College of Medicine (number: 1766). Informed consent was obtained from all of the participants.

## CRediT Author Statement

**Marumo Mikio:** Data collection, Validation, Reviewing; **Kazumi Ekawa:** Data collection, Validation; **Ichiro Wakabayashi:** Conceptualization, Data analysis, Writing.

## Declaration of Competing Interest

The authors declare that there are no competing interests regarding this article.

## Appendix Supplementary Materials

Image, application 1
